# Neuroprotective Effects of Phosphodiesterase Inhibitors on Sestrin-2 (SESN2) Expression and Autophagy in Alzheimer’s Disease Model

**DOI:** 10.7759/cureus.88449

**Published:** 2025-07-21

**Authors:** Gokhan Faikoglu, Kübra Saygisever-Faikoglu, Hande Ozbasak, Sedat Askin Ugur, Tugce Uskur, Ahmet Gökhan Akkan, Pelin Kelicen-Ugur, Sibel Ozyazgan

**Affiliations:** 1 Pharmacology, Istanbul University-Cerrahpaşa, Cerrahpaşa Faculty of Medicine, Istanbul, TUR; 2 Pharmacology, Centre for Experimental Medicine of the Slovak Academy of Sciences, Bratislava, SVK; 3 Pharmacology, Turkish Ministry of Health, Center of Public Health, Ankara, TUR; 4 Pharmacology, Kirklareli University Faculty of Medicine, Kirklareli, TUR; 5 Pharmacology, Bezmialem Vakıf University, Faculty of Medicine, Istanbul, TUR; 6 Pharmacology, Hacettepe University, Faculty of Pharmacy, Ankara, TUR

**Keywords:** alzheimer's disease, ampk, autophagy, mtor, phosphodiesterase inhibitors, sestrin-2

## Abstract

Objective: Alzheimer's disease (AD) is a progressive neurodegenerative disorder characterized by cognitive decline and the accumulation of amyloid-beta (Aβ) peptides. The neuroprotective protein sestrin-2 (SESN2) has been implicated in the cellular response to oxidative stress and autophagy, processes that are disrupted in AD. This study explores the effects of phosphodiesterase inhibitors (PDEIs) roflumilast (RF), rolipram (ROL), and tadalafil (TAD) on SESN2 expression and autophagy in Aβ25-35-treated hippocampal neuron (HT-22) cell cultures.

Methods: The HT-22 cells were exposed to 5 μM Aβ25-35 for 32 hours to induce AD-like pathology. Concurrently, cells were treated with PDEIs (ROL: 10 μM, TAD: 1.53 nM, RF: 5 μM). The SESN2, autophagy-related proteins (ATG5, beclin-1 (BECN1), LC3II), adenosine monophosphate-activated protein kinase (AMPK), and mTOR expression levels were analyzed using reverse transcription-quantitative polymerase chain reaction (RT-qPCR) and western blot techniques.

Results: The Aβ25-35 exposure significantly increased SESN2 expression and altered the levels of autophagy-related proteins, resulting in decreased active AMPK (phosphorylated (p)-AMPK) and increased active mTOR (phosphorylated (p)-mTOR). Treatment with PDEIs reduced the elevated SESN2 expression and modulated autophagy-related protein levels, enhancing ATG5, BECN1, and LC3II expression. The PDEIs also restored p-AMPK levels and reduced p-mTOR expression in Aβ25-35-treated cells.

Conclusion: The PDEIs exhibit neuroprotective effects in an in vitro AD model by reducing SESN2 overexpression and modulating autophagy through the AMPK/mTOR pathway. These findings suggest that PDEIs could be potential therapeutic agents for AD, targeting SESN2 and autophagy pathways to mitigate neurodegenerative damage.

## Introduction

Alzheimer's disease (AD) is the most common form of dementia, characterized by the accumulation of amyloid-beta (Aβ) peptides and the formation of neurofibrillary tangles in the brain, accompanied by neuronal loss, inflammation, and reduced cognitive functions. As of 2019, it was reported in the World Alzheimer's Report that there are over 70 million dementia patients worldwide, two-thirds of whom have AD [1-4].

Since Alois Alzheimer's first diagnosis of the disease in 1907, no definitive treatment for AD has been found in the 112 years that have passed. Epidemiological studies indicate that AD is a multifactorial disease. Age-related neuronal, central, and vascular disorders play a significant role in its development. Individuals carrying the ε4 allele of the ApoE gene, which codes for the apolipoprotein involved in cholesterol transport in the brain, are found to be at three to 15 times higher risk of developing AD [5]. Additionally, the presence of cardiovascular risk factors such as hypertension, dyslipidemia, and diabetes in patients has been shown to increase the risk of AD [6]. The drug groups approved by the Food and Drug Administration (FDA) for AD treatment, including cholinesterase inhibitors and N-methyl-D-aspartate (NMDA) receptor antagonists or their combinations, only provide temporary and symptomatic relief while causing severe side effects [7].

Due to AD being the most common type of dementia, it is likely that AD will be encountered more frequently in the near future. Therefore, it is essential to fully elucidate the pathophysiological mechanisms of the disease and identify therapeutic targets in this light. In AD, Aβ accumulation and tau phosphorylation resulting in neurofibrillary tangle formation are thought to be the initiating pathophysiological events of neurodegenerative damage [6]. In other words, AD is triggered by an increase in the synthesis and/or a decrease in the degradation of neurodegenerative protein aggregates. Based on this information, approaches that reduce Aβ accumulation or toxicity and enhance its degradation may prevent or slow the development of AD [1].

Current studies report that autophagy is crucial for the lysosomal degradation and clearance of protein aggregates like Aβ, which constitute the pathophysiology of AD [8-12]. Autophagy is necessary for the enzymatic degradation and removal of misfolded neurodegenerative proteins and damaged organelles, such as Aβ peptides [13]. Excessive accumulation of Aβ in brain tissues can lead to oxidative stress, damaging many intracellular organelles. Autophagy plays a neuroprotective role by ensuring the controlled removal of damaged organelles and macromolecules [14].

The nuclei and mitochondrial DNA of the affected brain regions in Alzheimer's patients are damaged [15]. Sestrins (SESN), which increase under various stress conditions such as genotoxic and oxidative stress, are proteins that suppress oxidative stress [16]. Sestrin-2 (SESN2) is the most studied of the three sestrin isoforms expressed in mammalian cells and is shown to be responsible for its cell-protective activity, antioxidant scavenging of free radicals, and autophagy-inducing activities [17,18]. The SESN2 is induced under stress conditions [19] and mediates autophagy induction by inhibiting mTOR through AMPK activation [20,21]. It is known that Aβ increases SESN2 in primary rat cortical cell cultures, activating antioxidant and autophagy pathways. These data indicate the relationship between SESN2 induction or inhibition and AD, a neurodegenerative disease. The SESN2, induced by p53 under stress conditions, plays a role in autophagy induction via mTOR inhibition by AMPK activation [22].

Studies have shown that exposure to Aβ increases SESN2 expression in different neuronal cell cultures [8,10], activating antioxidant and autophagy pathways. In a transgenic AD mouse model, a simultaneous increase in SESN2 expression and the autophagy marker LC3B-II was observed in the brain cortex. These findings demonstrate the close relationship between SESN2 induction or inhibition and AD, emphasizing the role of autophagy pathways in this relationship [23].

Recent studies emphasize the effects of phosphodiesterase inhibitors (PDEIs) on neurological and psychiatric diseases [24]. Phosphodiesterases (PDEs) are metallophosphohydrolase enzymes that metabolize cyclic adenosine monophosphate (cAMP) and cyclic guanosine monophosphate (cGMP), secondary messengers, into their inactive 5'-monophosphates [25]. The selective inhibition of cyclic nucleotide hydrolysis by PDEIs can be beneficial in various brain pathologies such as depression, schizophrenia, AD, and ischemia [26]. The effects of nonspecific PDEIs like resveratrol and specific PDEIs like cilostazol on AD disease models and autophagy support this hypothesis [23].

Our study aims to present SESN2, shown to increase protectively against neurodegenerative damage and Aβ toxicity and believed to be responsible for its antioxidant and autophagy-inducing mechanisms, as a new target for neuroprotective effects. In this context, the neuroprotective and autophagy-inducing pharmacological roles of PDEIs through SESN2 will be examined. *This article was previously presented as a meeting abstract at the 25th National Pharmacology Congress in 2019.*

## Materials and methods

This research was conducted at Atlas Biotechnology Laboratories (Ankara, TUR) in collaboration with Istanbul University-Cerrahpasa (Istanbul, TUR), Institute of Graduate Studies, Department of Medical Pharmacology (project no.: TDK-2018-30620).

Cell culture and protocol

Mouse hippocampal neurons (HT-22) sourced from Atlas Biotechnology were cultured in Dulbecco’s Modified Eagle Medium (DMEM) (Thermo Fisher Scientific, Waltham, MA, USA) with 10% fetal bovine serum (FBS), 1% L-glutamine, and penicillin/streptomycin (10,000 U/ml). The cultures were maintained at 37°C with 5% CO₂. To evaluate the effects of PDEIs on neuronal cells, various concentrations were tested: rolipram (RL) at 3, 10, and 30 μM; tadalafil (TAD) at 0.75, 1.53, and 3 nM; and roflumilast (RF) at 2.5, 5, and 10 μM over 32 hours. Subsequent treatments continued with selected concentrations: 1.53 μM for TAD, 5 μM for RF, and 10 μM for RL, along with Aβ25-35 (5 μM, 32 hours) as per established protocols. When cell confluency reached 70%, Aβ25-35 and PDEIs were applied, and cells were lysed after 32 hours. Prior to lysis, cell morphology and confluency were evaluated. Lysis was performed using ProteinEx Total Protein Extraction Solution (GeneAll, Seoul, KOR) with a Complete Protease Inhibitor Cocktail (Roche, Basel, CHE), dithiothreitol (DTT) (Amresco, Cleveland, OH, USA), and phenylmethylsulfonyl fluoride (PMSF) (Santa Cruz; Santa Cruz, CA, USA), mixed on ice. The lysates were centrifuged at 14,000 × g for 20 minutes at 4°C. Protein levels in the supernatants were determined using Qubit® Protein Assay Kits (Thermo Fisher Scientific). Cell lysates were stored at −80°C for future Western blot analysis.

Antibodies and pharmacological agents

This study utilized a range of primary antibodies and pharmacological agents. These included sirtuin 1 (SIRT1) and SESN2 antibodies from Bioassay Technology Laboratory (Shanghai, CHN); AMPK α1/2, phospho-AMPK α1/2 (Thr183/172), LC3B, mTOR, phospho-mTOR (Ser2448), and beta-actin (ACTB antibodies from Elabscience (Hubei, CHN). Other reagents included NuPAGE LDS Sample Buffer, NuPAGE Sample Reducing Agent, MES Running Buffer, and Novex™ ECL Chemiluminescent Substrate Reagent Kit from Thermo Fisher Scientific; Colour Protein Marker II and non-fat dry milk from Nzytech (Lisboa, PRT); iBlot Transfer Stack nitrocellulose kit, NuPAGE 4-12% Bis-Tris Gel, and Western Breeze Kit from Invitrogen (Carlsbad, CA, USA). Pharmacological inhibitors such as ibudilast, levosimendan (PDE3I), and vinpocetine (PDE1I) were obtained from Sigma Aldrich (St. Louis, MO, USA) along with Aβ25-35 human peptide (≥97% high-performance liquid chromatography (HPLC)). Additional cell culture components included DMEM, L-glutamine, trypsin ethylenediaminetetraacetic acid (EDTA), FBS heat-inactivated, and phosphate-buffered saline (PBS) from Capricorn Scientific (Ebsdorfergrund, DEU); penicillin/streptomycin from Biochrom AG (Berlin, DEU); bovine serum albumin (BSA), EDTA, Tris base, Tris hydrochloric acid (HCl), sodium dodecyl sulfate (SDS), and glycine from AppliChem (Darmstadt, DEU); protease inhibitor cocktail tablets from Roche (Basel, CHE); DTT from Amresco; sodium chloride (NaCl) and Tween-20 from Merck (Rahway, NJ, USA); methanol from Kimetsan (Ankara, TUR); and MTT dissolved in dimethyl sulfoxide (DMSO) from Sigma Aldrich.

Preparation of Aβ peptides

The Aβ25-35 human peptide, known as the toxic fragment of the complete Aβ1-42 peptide, was dissolved to a concentration of 1 mg/ml in sterile distilled water. For aggregation, this unaggregated peptide was incubated at 37°C for 72 hours with periodic gentle mixing. This solution was freshly prepared 72 hours before use to ensure maximum activity for experiments.

MTT reduction assay

The viability of HT-22 cells was assessed using the MTT reduction assay. The MTT was prepared in DMSO to form a 50 mg/mL stock solution, a concentration 100 times greater than typically used. Cells were seeded in 96-well plates and cultured for 12 hours before treatment with various concentrations of PDEIs, either alone or with Aβ25-35, for 32 hours. Following treatment, the HT-22 cells were incubated with 0.5 mg/mL MTT in the dark at 37°C for four hours, allowing viable cells to convert MTT into insoluble formazan. Each well then received 150 μL of isopropyl alcohol to dissolve the formazan crystals, followed by five minutes of agitation. The optical density (OD) of each solution was measured using a microplate spectrophotometer at 570 nm (Biotek Instruments Inc., Winooski, VT, USA).

Assessment of SIRT1, SESN2, ATG5, and BECN1 gene expression using quantitative real-time PCR (qRT-PCR)

Gene expression levels of SIRT1, SESN2, autophagy-related protein 5 (ATG5), and beclin-1 (BECN1) were analyzed using qRT-PCR. Total RNA was isolated using the Total RNA Isolation System (Nzytech), with purity confirmed spectrophotometrically by the 260/280 nm absorbance ratio. The RNA was reverse transcribed to cDNA using an RT-PCR kit (Strata Gene, La Jolla, CA, USA). The qRT-PCR was conducted with the SYBR Green Jump Start Taq ReadyMix (Sigma-Aldrich) per the manufacturer’s thermal cycling conditions. The ACTB was used as the internal reference. Expression results were quantified as fold changes relative to the control using the 2^-ΔΔCT method.

Western blot analysis

Cellular protein extracts for western blot analysis were prepared. Protein samples were prepared by diluting with 4X Sample Buffer (5 μl) and 10X NuPAGE Sample Reducing Agent (2 μl) from Thermo Fisher Scientific. Samples were boiled, and 50 μg of protein per lane was loaded onto a 4% to 12% bis-Tris gradient gel. Proteins were resolved via electrophoresis and transferred onto a nitrocellulose membrane using the iBlot Transfer Stack nitrocellulose kit (Thermo Fisher Scientific). Membranes were blocked with 5% non-fat dry milk in Tris-buffered saline (TBS; pH 7.4) for two hours, then incubated overnight at 4°C with rabbit primary antibodies. Both primary and secondary antibodies were diluted in TBS-T buffer (0.05% Tween-20, 0.2 M NaCl in 20 mM Tris-HCl, pH 7.5) with either 5% non-fat milk or 5% BSA. Membranes were treated for one hour at room temperature with horseradish peroxidase (HRP)-conjugated secondary antibodies in 5% non-fat dry milk or 5% BSA in TBS (pH 7.4). Immunoreactive bands were detected using a chemiluminescence system and documented using the GEN-BOX Imager CFX (ER Biotech Ltd., Ankara, TUR). Optical density measurements were performed using ImageJ 1.47 software (National Institutes of Health, Bethesda, MD, USA), and protein levels were normalized against ACTB (dilution 1:7500).

Statistical analysis

Multiple group comparisons were evaluated using two-way ANOVA and Tukey tests with GraphPad Prism 7.0 software (GraphPad Software Inc., La Jolla, CA, USA). Results are presented as mean ± standard error of the mean (SEM). A p-value of less than 0.05 (*p < 0.05) was considered statistically significant.

## Results

**Figure 1:**
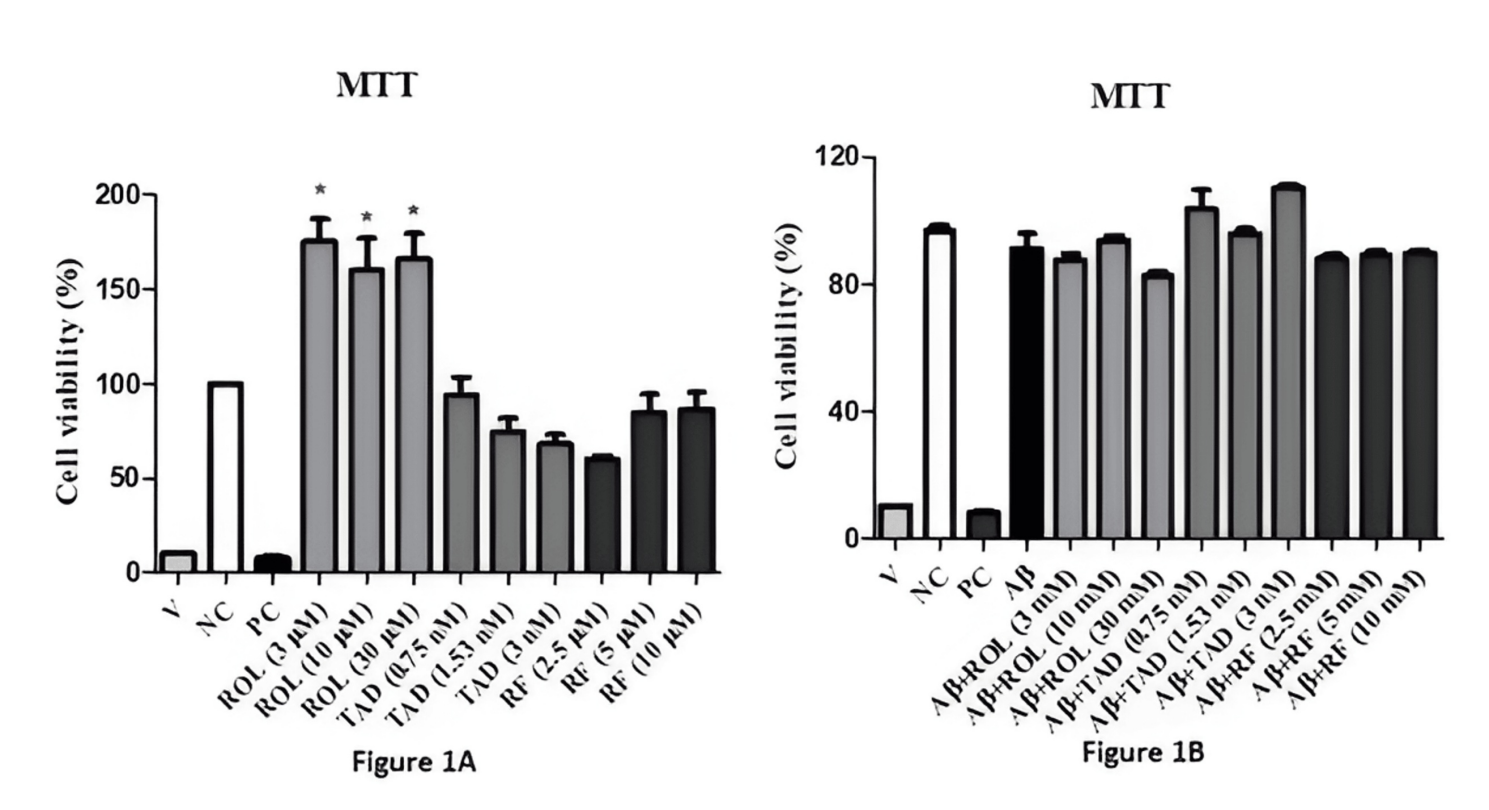
Evaluation of cytotoxicity results ROL: Rolipram, TAD: Tadalafil, RF: Roflumilast

Mouse hippocampal cell lines (HT-22) were incubated for 32 hours with different concentrations of PDEIs (rolipram (ROL): 3 μM, 10 μM, 30 μM; tadalafil (TAD): 0.75, 1.53 nM, 3 nM; roflumilast (RF): 2.5 μM, 5 μM, 10 μM) and Aβ25-35 at a concentration of 5 μM. Cell viability was measured using the MTT assay, comparing against negative and positive controls. All concentrations of ROL (3 μM, 10 μM, 30 μM) showed an increase in cell viability compared to the negative control (p<0.05; n=4). This suggests that ROL may have a cell proliferation-enhancing effect (Figure 1A). The TAD (0.75, 1.53 nM, 3 nM) and RF (2.5 μM, 5 μM, 10 μM) did not result in a significant decrease in cell viability compared to the negative control (Figure 1A; p>0.05; n=4). Application of Aβ25-35 alone and in combination with PDEIs did not cause a significant reduction in cell viability (Figure 1B; p>0.05; n=4). Similarly, treatment with Aβ25-35 at a concentration of 5 μM for 32 hours, both alone and in combination with PDEIs, did not significantly reduce cell viability (Figure 1B; p>0.05; n=4).

**Figure 2:**
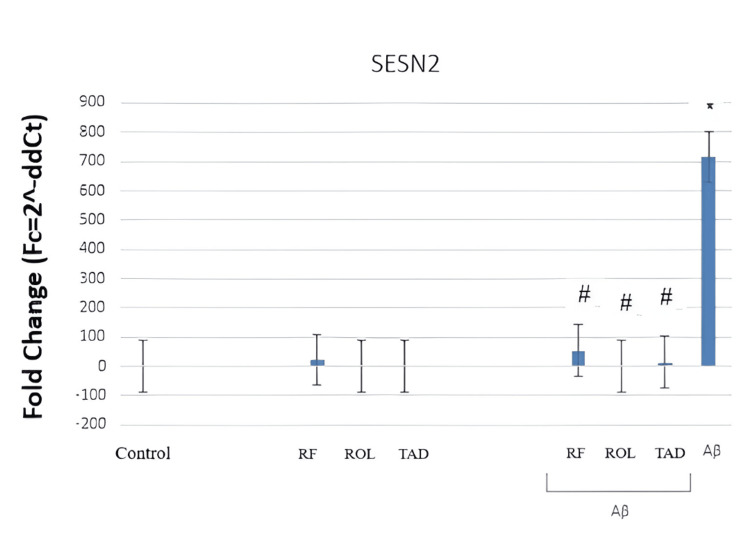
RT-qPCR graph showing the effects of Aβ25–35 and/or simultaneous PDEI treatment on SESN2 gene expression in HT-22 RT-qPCR: Reverse transcription-quantitative polymerase chain reaction, PDEI: Phosphodiesterase inhibitor, SESN2: Sestrin-2, ROL: Rolipram, TAD: Tadalafil, RF: Roflumilast, HT-22: Mouse hippocampal neuron cells

The graph (Figure 2) illustrates the effects of Aβ25-35 (5 μM) application and/or simultaneous PDEI treatment (ROL: 10 μM; TAD: 1.53 nM; RF: 5 μM) on SESN2 gene expression in HT-22 cells. Quantification of mRNA expression was normalized using the ACTB transcript as a reference. The cells were exposed to 5 μM Aβ25-35 for 32 hours and/or co-treated with 5 μM Aβ25-35 and PDEIs (ROL: 10 μM; TAD: 1.53 nM; RF: 5 μM). Control groups were incubated with 0.1% DMSO.

The incubation of cells with 5 μM Aβ25-35 for 32 hours increased SESN2 gene expression. While RF (5 μM), ROL (10 μM), and TAD (50 μM) alone maintained SESN2 expression at control levels, their combined application with Aβ25-35 brought the elevated SESN2 expression back to control values (Figure 2). In summary, RT-qPCR results indicate that 5 μM Aβ25-35 (32 hours) led to an increase in SESN2 gene expression (*p<0.05). However, simultaneous treatment with PDEIs and Aβ25-35 reduced the elevated SESN2 expression (^#^p<0.05) (Figure 2).

**Figure 3:**
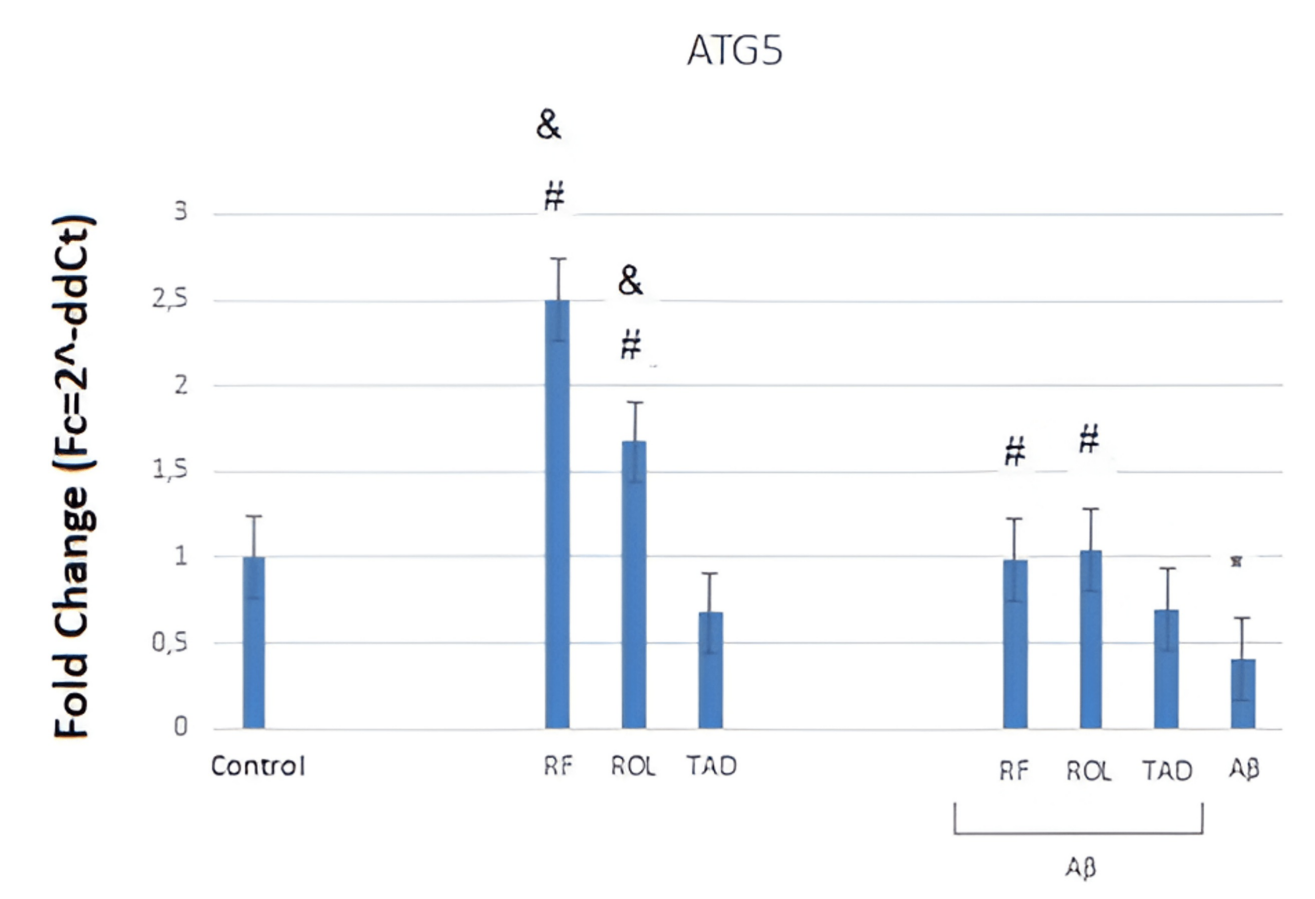
RT-qPCR graph showing the effects of Aβ25–35 and/or simultaneous PDEI treatment on ATG5 gene expression in mouse hippocampal neuron cells RT-qPCR: Reverse transcription-quantitative polymerase chain reaction, PDEI: Phosphodiesterase inhibitor, ROL: Rolipram, TAD: Tadalafil, RF: Roflumilast, HT-22: Mouse hippocampal neuron cells

The graph (Figure 3) illustrates the effects of Aβ25-35 (5 μM) application and/or simultaneous PDEI treatment (ROL: 10 μM; TAD: 1.53 nM; RF: 5 μM) on ATG5 gene expression in HT-22 cells. Quantification of mRNA expression was normalized using the ACTB transcript as a reference. The cells were exposed to 5 μM Aβ25-35 for 32 hours and/or co-treated with 5 μM Aβ25-35 and PDEIs (ROL: 10 μM; TAD: 1.53 nM; RF: 5 μM). Control groups were incubated with 0.1% DMSO. Data were obtained from a single experiment (Figure 3).

Incubation of cells with 5 μM Aβ25-35 for 32 hours resulted in a decrease in the expression of the autophagy-related ATG5 gene. The RF (5 μM) and ROL (10 μM) alone increased ATG5 expression compared to the control (*p<0.05). When co-applied with Aβ25-35, they restored the decreased ATG5 expression to control levels (#p<0.05). The TAD (50 μM) alone did not alter ATG5 expression compared to the control, nor did it change ATG5 expression when co-applied with Aβ25-35 (Figure 3). In summary, the application of 5 μM Aβ25-35 for 32 hours reduced ATG5 gene expression compared to the control (*p<0.05). Simultaneous treatment with RF (5 μM) and ROL (10 μM) with Aβ25-35 increased the reduced ATG5 gene expression (^#^p<0.05). The RF (5 μM) and ROL (10 μM) alone caused an increase in ATG5 gene expression compared to TAD (50 μM) (^&^p<0.05) (Figure 3).

**Figure 4:**
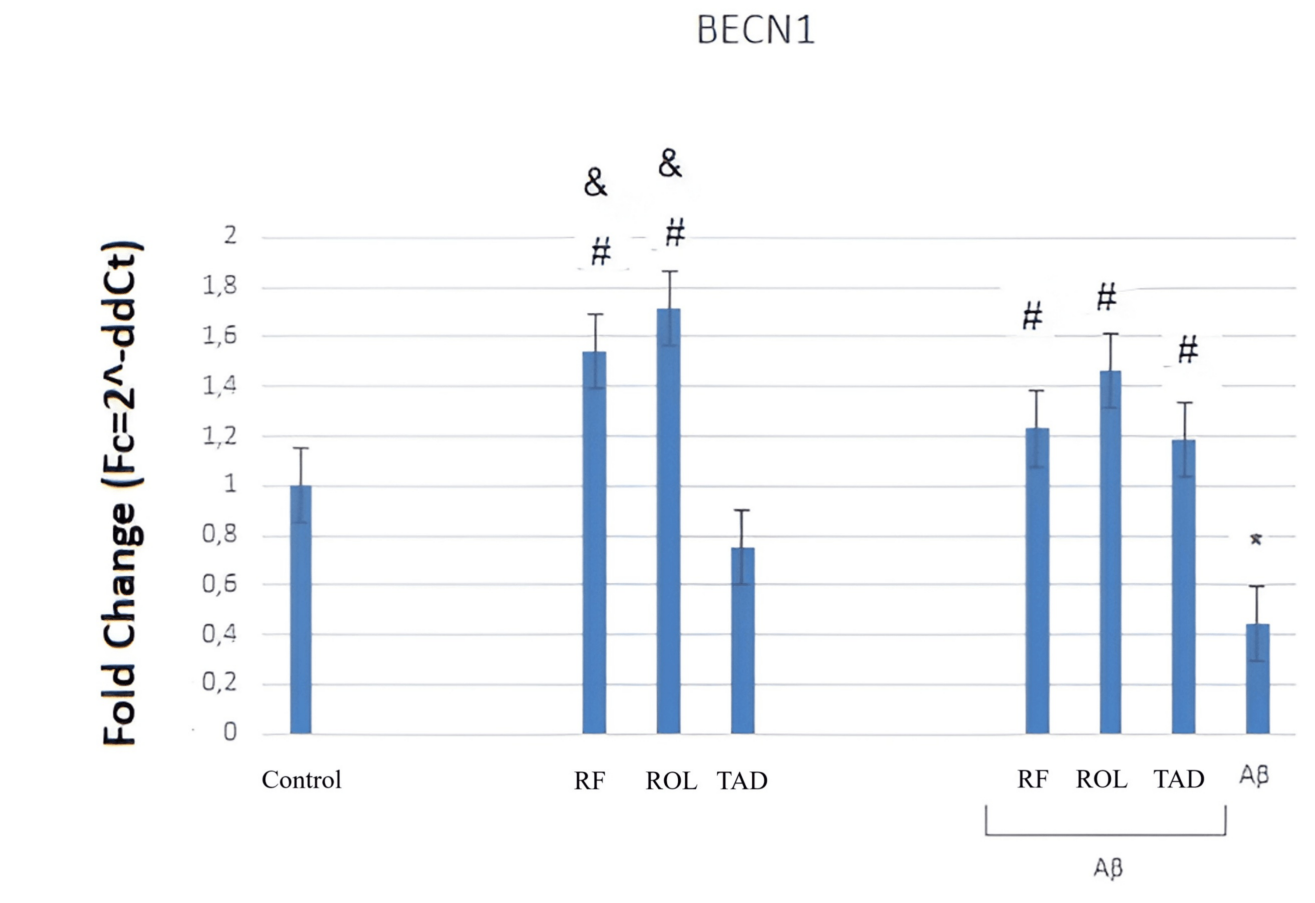
Effects of PDEIs and Aβ25-35 on BECN1 gene expression PDEI: Phosphodiesterase inhibitor, ROL: Rolipram, TAD: Tadalafil, RF: Roflumilast, BECN1: Beclin-1

The RT-qPCR graph (Figure 4) shows the effects of Aβ25-35 (5 μM) application and/or simultaneous PDEI treatment (ROL: 10 μM; TAD: 1.53 nM; RF: 5 μM) on BECN1 gene expression in HT-22 cells. The quantification of mRNA expression was normalized using the ACTB transcript as a reference. Cells were exposed to 5 μM Aβ25-35 for 32 hours and/or treated with 5 μM Aβ25-35 along with PDEIs (ROL: 10 μM; TAD: 1.53 nM; RF: 5 μM). Control groups were incubated with 0.1% DMSO. The data were obtained from a single experiment (Figure 4).

Incubation of cells with 5 μM Aβ25-35 for 32 hours resulted in a decrease in the expression of the autophagy-related BECN1 gene. The ROL (10 μM) and RF (5 μM) alone increased BECN1 expression compared to the control, while TAD (50 μM) alone did not change BECN1 gene expression. The simultaneous application of all three PDEIs with Aβ25-35 prevented the decrease in BECN1 expression caused by Aβ25-35 (Figure 4).

In summary, the application of 5 μM Aβ25-35 for 32 hours reduced BECN1 gene expression (*p<0.05). Simultaneous treatment with PDEIs and Aβ25-35 increased the reduced BECN1 expression (^#^p<0.05). The RF (5 μM) and ROL (10 μM) alone caused a greater increase in BECN1 gene expression compared to TAD (50 μM) (&p<0.05) (Figure 4).

**Figure 5:**
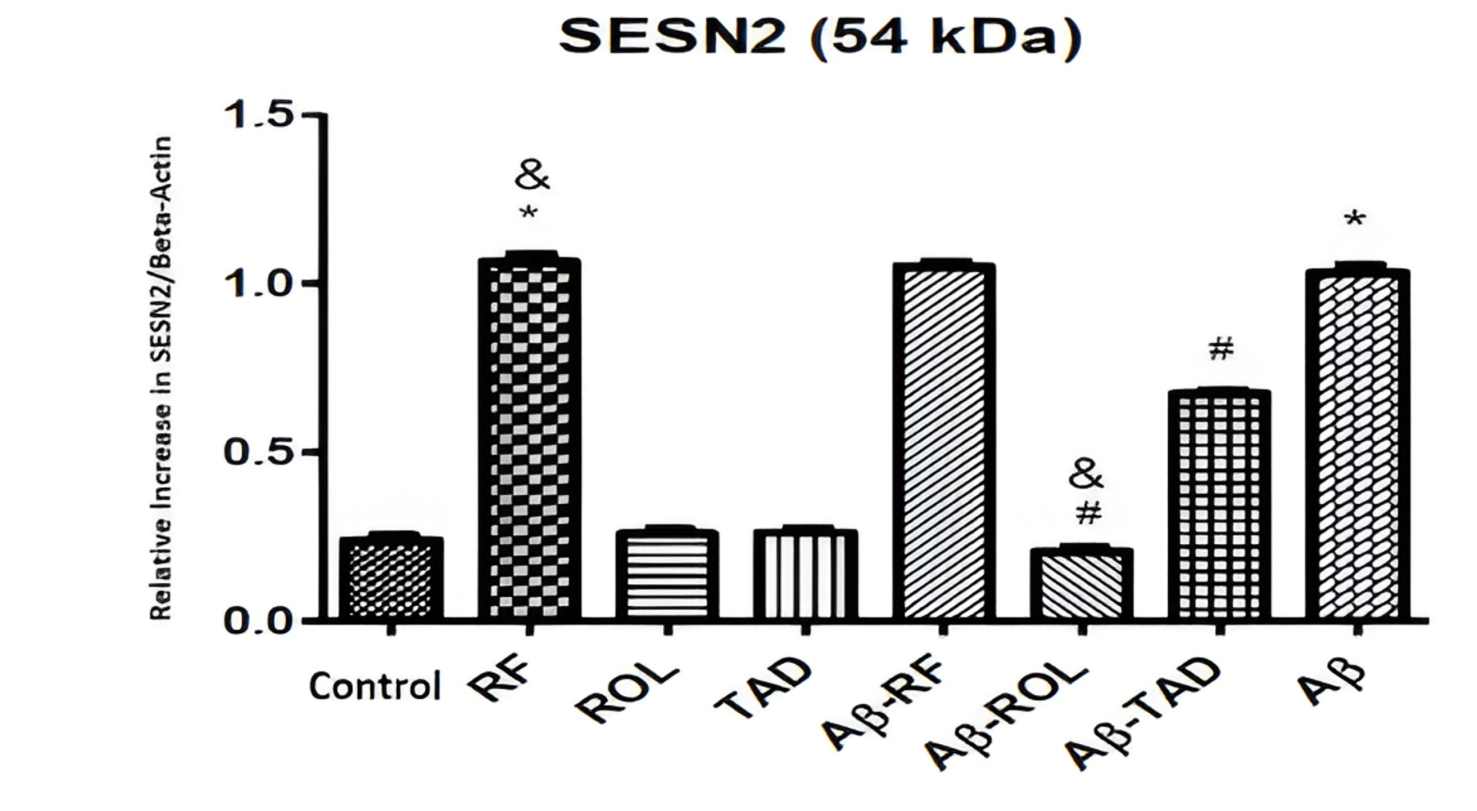
Effects of PDEI and Aβ25-35 application on SESN2 protein expression PDEI: Phosphodiesterase inhibitor, SESN2: Sestrin-2, ROL: Rolipram, TAD: Tadalafil, RF: Roflumilast

The Western blot analysis demonstrates the effects of Aβ25-35 (5 μM) application and/or simultaneous PDEI treatment (ROL: 10 μM; TAD: 1.53 nM; RF: 5 μM) on SESN2 protein expression in HT-22 cells, normalized to β-actin. Cells were exposed to 5 μM Aβ25-35 for 32 hours and/or co-treated with PDEIs (ROL: 10 μM; TAD: 1.53 nM; RF: 5 μM), with control groups incubated with 0.1% DMSO (Figure 5).

Data expressed as mean ± SEM from three independent experiments indicate that Aβ25-35 significantly increased SESN2 expression (*p<0.05). ROL (10 μM) and TAD (1.53 nM) alone did not significantly alter SESN2 levels compared to control, but reduced the SESN2 increase when combined with Aβ25-35 (#p<0.05). The RF (5 μM) alone significantly increased SESN2 expression (*p<0.01) and did not change the SESN2 increase when combined with Aβ25-35, suggesting a direct effect of RF on SESN2, consistent with RT-qPCR results (Figure 5).

Additionally, ROL (10 μM) combined with Aβ25-35 caused a more significant reduction in SESN2 protein compared to TAD (1.53 nM) combined with Aβ25-35 (&p<0.01), and RF (5 μM) alone increased SESN2 expression (&p<0.01), an effect not seen with the other PDEIs alone (Figure 5).

**Figure 6:**
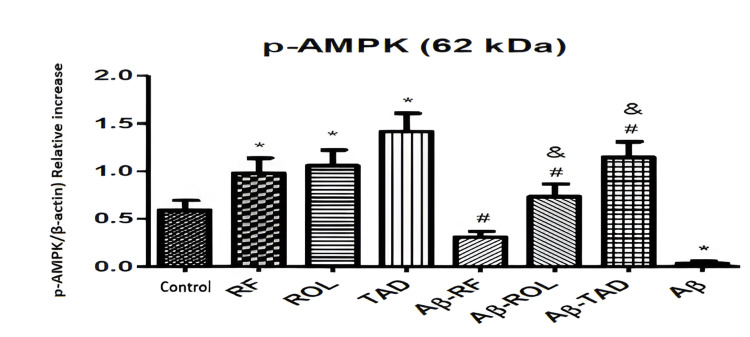
Effects of PDEIs and Aβ25-35 on p-AMPK protein expression PDEI: Phosphodiesterase inhibitor, SESN2: Sestrin-2, ROL: Rolipram, TAD: Tadalafil, RF: Roflumilast, p-AMPK: Phosphorylated AMPK

The Western blot analysis demonstrates the effects of Aβ25-35 (5 μM) application and/or simultaneous PDEI treatment (ROL: 10 μM; TAD: 1.53 nM; RF: 5 μM) on p-AMPK protein expression in HT-22 cells. The bands are normalized to the internal standard β-actin. Cells were exposed to 5 μM Aβ25-35 for 32 hours and/or treated with 5 μM Aβ25-35 along with PDEIs (ROL: 10 μM; TAD: 1.53 nM; RF: 5 μM). Control groups were incubated with 0.1% DMSO. Data are presented as the mean ± SEM from three independent experiments (*p<0.05 compared to the control group; ^#^p<0.05 compared to the 5 μM Aβ25-35 group) (Figure 6).

Incubation of cells with 5 μM Aβ25-35 for 32 hours resulted in a decrease in the expression of AMPK and its active form, p-AMPK, which are associated with cell proliferation and protection. The ROL (10 μM), RF (5 μM), and TAD (50 μM) alone increased p-AMPK levels compared to the control (*p<0.05) but did not change AMPK levels. Simultaneous application of PDEIs with Aβ25-35 prevented the decrease in AMPK and p-AMPK caused by Aβ25-35 (^#^p<0.05). Co-application of ROL (10 μM) and TAD (50 μM) with Aβ25-35 resulted in a more significant increase in p-AMPK compared to co-application of RF (5 μM) with Aβ25-35 (^&^p<0.05) (Figure 6).

**Figure 7:**
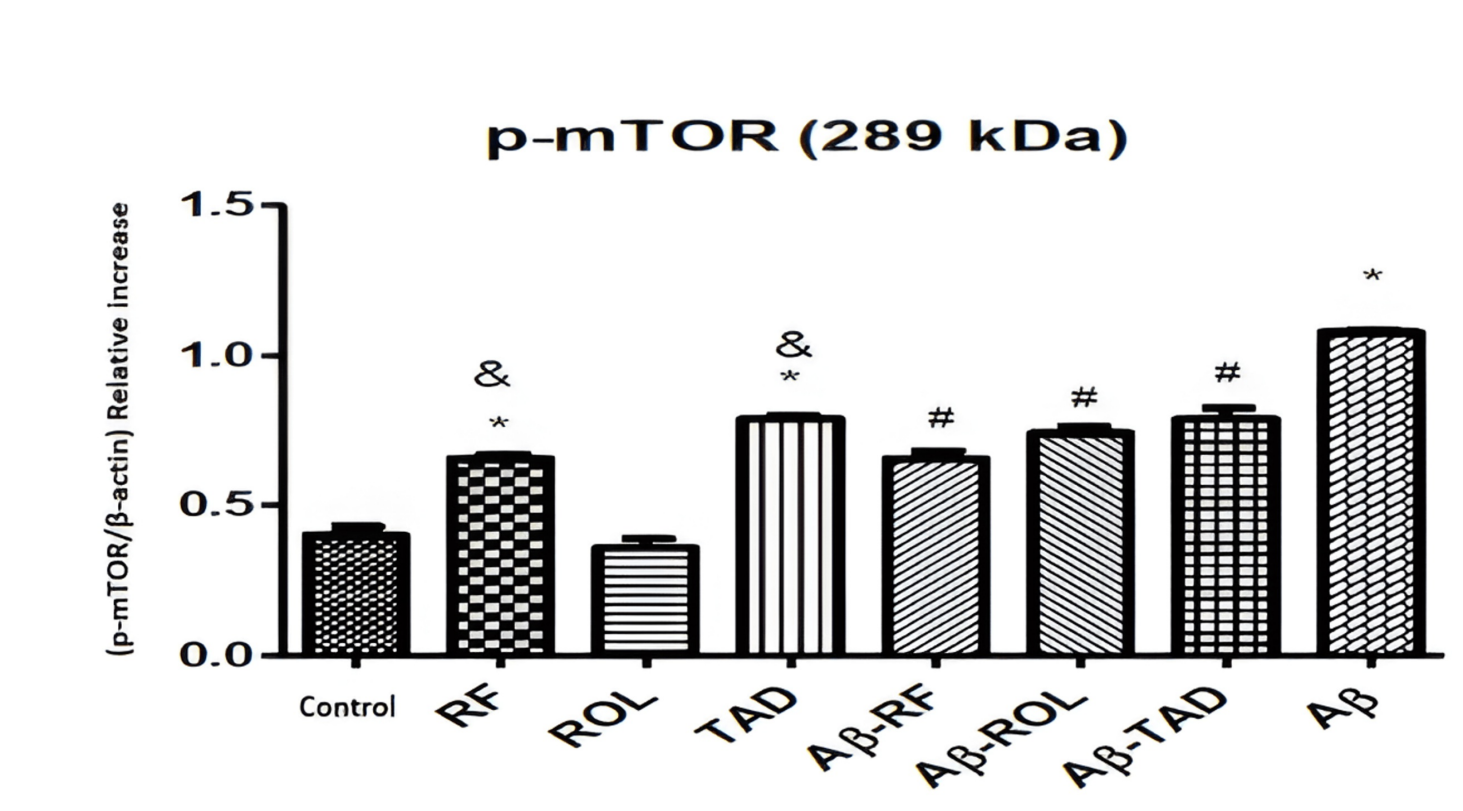
Effects of PDEI and Aβ25-35 application on mTOR protein expression PDEI: Phosphodiesterase inhibitor, ROL: Rolipram, TAD: Tadalafil, RF: Roflumilast, p-mTOR: Phosphorylated mTOR

The Western blot bands (A) and comparative mTOR densities (B) in Figure 7 illustrate the effects of Aβ25-35 (5 μM) application and/or simultaneous PDEI treatment (ROL: 10 μM; TAD: 1.53 nM; RF: 5 μM) on mTOR protein expression in HT-22 cells. The bands are normalized to the internal standard β-actin. Cells were exposed to 5 μM Aβ25-35 for 32 hours and/or treated with 5 μM Aβ25-35 along with PDEIs. Control groups were incubated with 0.1% DMSO. Data are presented as the mean ± SEM from three independent experiments (*p<0.05 compared to the control group; #p<0.05 compared to the 5 μM Aβ25-35 group) (Figure 7).

Incubation of cells with 5 μM Aβ25-35 for 32 hours resulted in an increase in the expression of mTOR and its active form, p-mTOR, which are associated with reduced cell proliferation and viability. RF (5 μM) and TAD (50 μM) alone increased p-mTOR levels compared to the control (*p<0.05) but did not change mTOR levels. Simultaneous application of PDEIs with Aβ25-35 significantly reduced the Aβ25-35-induced increase in p-mTOR (^#^p<0.05) without affecting mTOR levels. The RF (5 μM) and TAD (50 μM) alone caused a more significant increase in p-mTOR compared to ROL (10 μM) (^&^p<0.05) (Figure 7).

**Figure 8:**
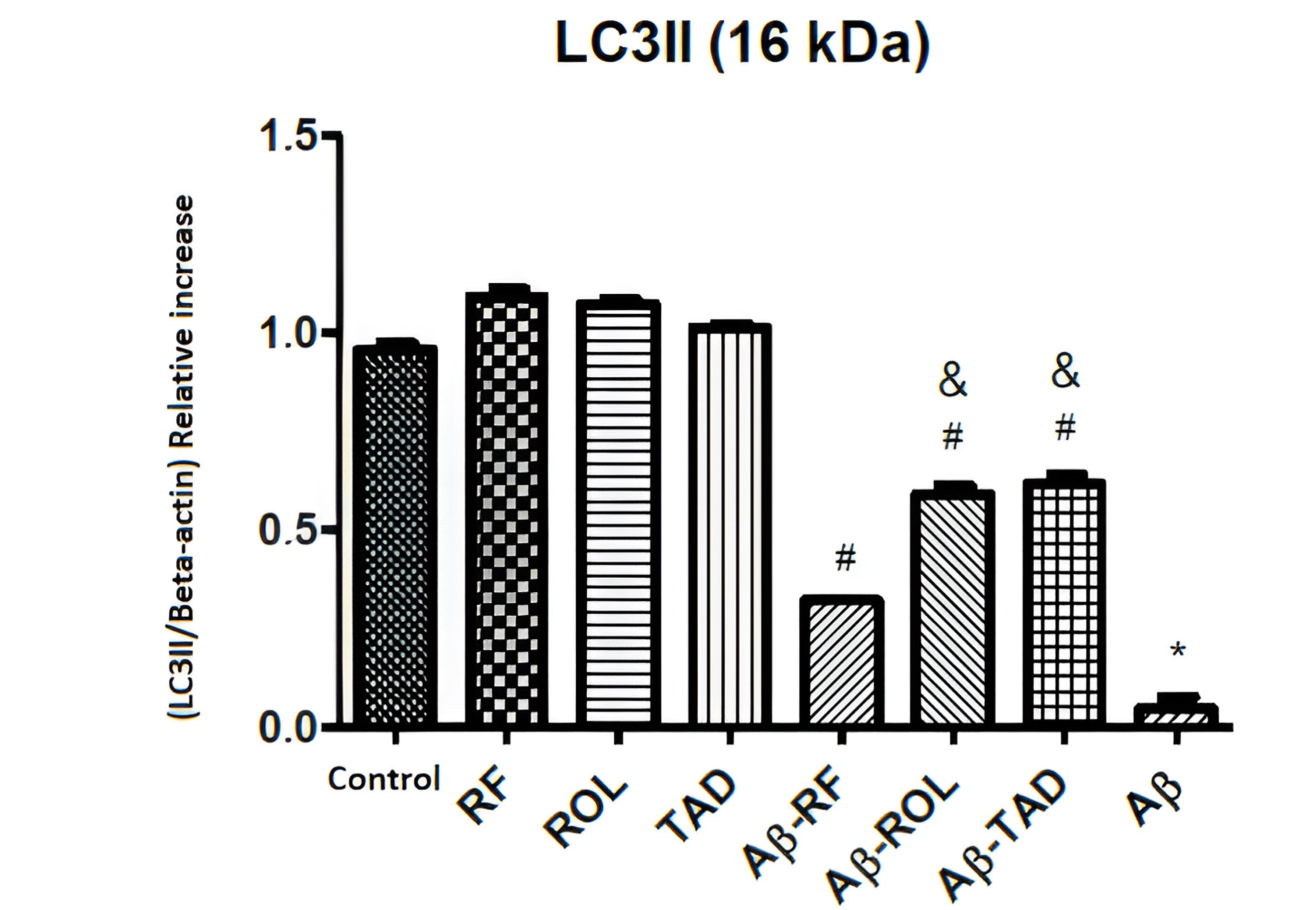
Effects of PDEI and Aβ25-35 application on LC3II protein expression PDEI: Phosphodiesterase inhibitor, ROL: Rolipram, TAD: Tadalafil, RF: Roflumilast

The western blot bands (A) and comparative LC3II densities (B) in Figure 8 illustrate the effects of Aβ25-35 (5 μM) application and/or simultaneous PDEI treatment (ROL: 10 μM; TAD: 1.53 nM; RF: 5 μM) on LC3II protein expression in HT-22 cells. The bands are normalized to the internal standard β-actin. Cells were exposed to 5 μM Aβ25-35 for 32 hours and/or treated with 5 μM Aβ25-35 along with PDEIs. Control groups were incubated with 0.1% DMSO. Data are presented as the mean ± SEM from three independent experiments (*p<0.05 compared to the control group; ^#^p<0.05 compared to the 5 μM Aβ25-35 group).

In HT-22 cells, LC3 was observed as a single band (II). Treatment with PDEIs alone (ROL: 10 μM, RF: 5 μM, TAD: 50 μM) did not alter LC3II protein expression, whereas Aβ25-35 significantly decreased LC3II expression (*p<0.05). However, the combined application of Aβ25-35 with PDEIs significantly increased the LC3II protein expression that was reduced by Aβ25-35 alone (#p<0.05). Additionally, the combination of ROL (10 μM) and TAD (50 μM) with Aβ25-35 resulted in a more significant increase in LC3II protein expression compared to the combination of RF (5 μM) with Aβ25-35 (&p<0.05) (Figure 8).

## Discussion

This study aims to demonstrate the effects of RF, ROL, and TAD on the neuroprotective protein SESN2 in HT-22 cell cultures treated with the neurotoxic protein Aβ25-35, which is responsible for the pathogenesis of AD. The findings show that 32-hour exposure to 5 μM Aβ25-35 in HT-22 cell cultures increased SESN2 expression. Concurrent application of PDEIs with Aβ25-35 reduced the increased SESN2 gene expression. The Aβ25-35 (5 μM; 32 hours) reduced the expression of autophagy-related proteins ATG5, BECN1, and LC3II, while simultaneous PDEI application increased their expression.

Alzheimer's disease is a significant progressive neurodegenerative disorder characterized by cognitive and behavioral deficiencies [27]. It is a complex disease involving multiple pathogenic pathways and changes in the expression of thousands of genes due to the influence of many genes and environmental factors. It is marked by the accumulation of Aβ in neurons, tau hyperphosphorylation, inflammation, oxidative stress, energy metabolism errors, and mistakes in the cell cycle and apoptosis [28]. The SESNs are highlighted as genes whose expression changes in AD. The SESNs have high antioxidant capacity [29], are upregulated under various stress conditions like genotoxicity and oxidative stress, and suppress oxidative stress [16]. Of the three SESN isoforms expressed in mammalian cells (SESN1, SESN2, SESN3) [30], SESN2 is the most studied and is responsible for cell protective effects, scavenging free radicals, and inducing autophagy [17,18,22]. Studies in primary rat cortical neuron cultures have shown that Aβ increases SESN2 expression, activating antioxidant and autophagy pathways. These findings indicate that SESN2 induction or inhibition is closely related to AD [22].

In our study, both RT-qPCR and western blot results indicated that Aβ25-35 (5 μM; 32 hours) increased SESN2 protein expression, aligning with the literature. Under stress conditions, SESN2 is induced by p53 and activates AMPK, which inhibits mTOR, leading to autophagy induction [20, 21]. This effect is achieved through AMPK activation, independent of redox regulatory activities. The SESN2-dependent mTOR inhibition is crucial for the autophagy-mediated degradation of proteins, inhibiting antioxidant genes. The AMPK activation allows SESN2 to inhibit enzymes producing pathogenic amounts of reactive oxygen species (ROS) [29]. The SESN antioxidant activities are also regulated by p53 and FoxO transcription factors. High levels of oxidative stress cause cell death via p53 and FoxO-dependent apoptotic gene transcription, while low levels stimulate SESNs, reducing oxidative stress and preventing cell death [31-33]. In our study, Aβ25-35 (5 μM; 32 hours) increased SESN2 expression but decreased active AMPK (phosphorylated (p)-AMPK) and increased active mTOR (phosphorylated (p)-mTOR) expression. We hypothesized that Aβ25-35-induced low-level oxidative stress is detected by cells, triggering compensatory SESN2 increases. The reduction in p-AMPK and the increase in p-mTOR expression with Aβ25-35 are likely direct effects of Aβ25-35.

Neurodegenerative diseases stem from an imbalance between protein production and degradation, leading to the accumulation of protein aggregates like Aβ and tau in AD. Lysosomal autophagy is a primary degradation system for these aggregates. Autophagy's neuroprotective roles include the controlled removal of damaged organelles and macromolecules, crucial for cellular homeostasis. Defects in these degradation mechanisms can contribute to neurodegenerative diseases like AD [34-38]. Many studies have shown a close relationship between AD pathology and autophagy pathways [8-12]. The AMPK activation, sensitive to cellular energy, inhibits the mTOR complex, inducing autophagy and enhancing neural energy status [39]. The mTOR is a key negative regulator of autophagy, and studies have shown hyperactive mTOR signaling in selected brain regions of AD patients, suppressing autophagy [40,41]. In a study with Tg2576 mice, reducing mTOR signaling and gene expression in the hippocampus increased autophagy and decreased Aβ accumulation, protecting against memory impairments [42]. In our study, 32-hour incubation with 5 μM Aβ25-35 decreased the expression of p-AMPK, lifting AMPK's inhibition of mTOR, which may increase the activity of the autophagy inhibitor mTOR (p-mTOR). Although Aβ25-35 increased SESN2 expression in HT-22 cells, it was insufficient to trigger p-AMPK increases, resulting in decreased p-AMPK and increased p-mTOR.

The effectiveness of lysosomal degradation mechanisms declines in AD, leading to the accumulation of cellular proteins and Aβ in the brain [43]. Autophagy is vital for removing Aβ aggregates and protecting healthy neurons from Aβ cytotoxicity. The transport of intracellular autophagosomes to lysosomes for degradation is called macroautophagy. Many studies report that macroautophagy is impaired in AD brains, leading to the accumulation of Aβ-containing autophagic vacuoles and increased neurodegenerative pathology [44]. Autophagy clears Aβ aggregates, protecting neurons from Aβ cytotoxicity. The formation of autophagosomes is regulated by mTOR and various autophagy-related proteins (Atg). The LC3B-I, a cytosolic Atg, converts post-translationally to LC3B-II during autophagosome formation [45]. The AMPK activation, a major regulator of metabolism, inhibits mTOR, inducing autophagy. Studies have shown that AMPK activators inhibit mTOR signaling, increasing autophagy and lysosomal Aβ degradation, while Aβ increases mTOR, and decreasing Aβ reduces mTOR [45-48]. In CHP 134 neuroblastoma cells, Aβ1-42, responsible for AD, increases SESN2 expression [8,10]. The Aβ increases SESN2 expression and activates antioxidant and autophagy pathways in primary rat cortical neuron cultures. In the transgenic AD mouse model APPswe/PSEN1dE9, increased SESN2 expression was observed in the cortex of 12-month-old mice. Simultaneous increases in SESN2 and the autophagosome marker LC3B-II were also observed in this model and primary cortical neuron cultures. The SESN2 siRNA reversed the Aβ-induced SESN2 increase and the associated LC3B-II decrease. These studies highlight a close relationship between neuroprotective and autophagy pathways in diseases like AD.

We investigated the potential of SESN2 as a new neuroprotective target and examined its relationship with PDEIs, which are known for their protective effects in neurodegenerative diseases. Initial cytotoxicity studies were conducted to determine the effects of PDEIs on cell viability and to establish appropriate concentrations. The PDEIs increased cell viability across all concentrations tested, likely due to their proliferative effects on cells.

The neuroprotective effects of different PDEIs have been reported in the literature. Based on these findings, we examined the effects of two PDE4 inhibitors and one PDE5 inhibitor on SESN2, BECN1, ATG5, and LC3-II expression in an in vitro AD model. This study provides important insights into the molecular mechanisms of AD by demonstrating the neuroprotective effects of PDE4 and PDE5 inhibitors on SESN2 and Atg. The findings demonstrate the potential of these inhibitors to slow or attenuate the progression of AD pathology.

This study has several important limitations. First, the in vitro model used may not fully reflect the complex pathological processes of AD, potentially limiting the direct applicability of the findings to clinical settings. Additionally, only the HT-22 murine hippocampal neuronal cell line was used in this study; further research involving different cell types or in vivo models could enhance the generalizability of the results. Furthermore, the long-term effects and dose-dependent side effects of PDEIs were not assessed in this study. Future research could provide more comprehensive insights into the prolonged use and molecular mechanisms of these compounds.

## Conclusions

The results indicate that PDEIs exert pharmacological effects in Aβ25-35-treated cells by reducing increased SESN2 expression and effectively inhibiting mTOR, thereby supporting cell protection through AMPK activation and autophagy regulation. The reduction of SESN2 with PDEI application suggests that PDEIs provide protective effects without compensating for SESN2. The increase in autophagy proteins ATG5, beclin-1, and LC3II with PDEI treatment indicates that PDEIs enhance protective and autophagy-inducing mechanisms in cells, although each PDEI acts on different components of autophagy. Future studies should evaluate the effects of these compounds at different dosages and over long periods in in vivo models, and their efficacy and safety profiles should be investigated more extensively. In addition, personalized treatment strategies can be developed by targeting SESN2 and autophagy pathways at the molecular level. In this way, it can contribute to the development of new and effective pharmacological approaches in the treatment of AD.
